# Smoking-Induced Disturbed Sleep. A Distinct Sleep-Related Disorder Pattern?

**DOI:** 10.3390/healthcare11020205

**Published:** 2023-01-10

**Authors:** Ioanna Grigoriou, Paschalia Skalisti, Ioanna Papagiouvanni, Anastasia Michailidou, Konstantinos Charalampidis, Serafeim-Chrysovalantis Kotoulas, Konstantinos Porpodis, Dionysios Spyratos, Athanasia Pataka

**Affiliations:** 1 Respiratory Failure Clinic, Papanikolaou General Hospital, Aristotle University of Thessaloniki, 57010 Thessaloniki, Greece; 2 Fourth Department of Internal Medicine, Hippokration General Hospital, Aristotle University of Thessaloniki, 54642 Thessaloniki, Greece; 3 Second Propaedeutic Department of Internal Medicine, Hippokration General Hospital, Aristotle University of Thessaloniki, 54642 Thessaloniki, Greece; 4 ICU, Hippokration General Hospital, Aristotle University of Thessaloniki, 54642 Thessaloniki, Greece; 5 Pulmonology Department, Papanikolaou General Hospital, Aristotle University of Thessaloniki, 57010 Thessaloniki, Greece

**Keywords:** obstructive sleep apnea, smoking, smoking-induced disturbed sleep pattern, obstructive sleep apnea–hypopnea

## Abstract

The relationship between smoking and sleep disorders has not been investigated sufficiently yet. Many aspects, especially regarding non-obstructive sleep apnea–hypopnea (OSA)-related disorders, are still to be addressed. All adult patients who visited a tertiary sleep clinic and provided information about their smoking history were included in this cross-sectional study. In total, 4347 patients were divided into current, former and never smokers, while current and former smokers were also grouped, forming a group of ever smokers. Sleep-related characteristics, derived from questionnaires and sleep studies, were compared between those groups. Ever smokers presented with significantly greater body mass index (BMI), neck and waist circumference and with increased frequency of metabolic and cardiovascular co-morbidities compared to never smokers. They also presented significantly higher apnea–hypopnea index (AHI) compared to never smokers (34.4 ± 24.6 events/h vs. 31.7 ± 23.6 events/h, *p* < 0.001) and were diagnosed more frequently with severe and moderate OSA (50.3% vs. 46.9% and 26.2% vs. 24.8% respectively). Epworth sleepiness scale (ESS) (*p* = 0.13) did not differ between groups. Ever smokers, compared to never smokers, presented more frequent episodes of sleep talking (30.8% vs. 26.6%, *p* = 0.004), abnormal movements (31.1% vs. 27.7%, *p* = 0.021), restless sleep (59.1% vs. 51.6%, *p* < 0.001) and leg movements (*p* = 0.002) during sleep. Those were more evident in current smokers and correlated significantly with increasing AHI. These significant findings suggest the existence of a smoking-induced disturbed sleep pattern.

## 1. Introduction

Smoking is now considered a chronic relapsing disease, which is rather difficult to treat. According to the World Health Organization (WHO), smoking is a major cause of early death worldwide, responsible not only for health problems, but also for increasing the costs to healthcare systems [1]. Smoking-related diseases, such as ischemic heart disease, ischemic cerebral disease and lower respiratory tract diseases are responsible for a great proportion of deaths worldwide (12.9%, 11.4% and 5.9%, respectively) [2]. The relative risk attributed to smoking is estimated to be 26.7% for ischemic heart disease, 32.2% for ischemic cerebral disease and 72.2% for chronic obstructive pulmonary disease (COPD) [3].

Obstructive sleep apneas–hypopneas (OSA) affect 17% and 9% of middle-aged males and females, respectively [4], while obstructive sleep apnea–hypopnea syndrome (OSAHS), which is characterized by symptoms, such as excessive daytime sleepiness, affects 3–7% of the population worldwide [5]. OSAHS symptoms include excessive daytime sleepiness, snoring, non-refreshing sleep, gasping–choking episodes and awakenings during sleep. OSA can emerge in every sleep stage; however, the respiratory events are more often during rapid eye movement (REM) sleep, due to the decreased muscle tone. Dynamic narrowing of the upper airways during sleep causes repeated apneic episodes, leading to sleep fragmentation and excessive daytime sleepiness [6]. Additionally, repeated sleep apneas result in hypoxemia, hypercarbia, hypertension and increased sympathetic tone, increasing the risk of endothelial dysfunction [7]. Significant intrathoracic pressure swings and increased blood pressure during sleep are considered to augment the risk for cardiovascular events in patients with OSA [8]. Therefore, OSA, along with smoking, both constitute significant risk factors for cardiovascular disease.

Many studies have tried to prove the relationship between OSAHS and smoking, showing a higher prevalence of smoking in OSAHS patients [9,10]. Additionally, there is evidence that smoking might be a risk factor for apneas and snoring [11,12,13]. Smokers present decreased sleep quality with greater sleep latency and increased difficulty in maintaining sleep [14,15,16]. Smoking worsens chronic airway inflammation, contributing to OSAHS symptoms [17]. Active and passive smoking, as well as a history of smoking, have been correlated with snoring [12]. Nevertheless, evidence is still conflicting, failing to prove a definite and clinically significant correlation between smoking and OSAHS. Despite that, possible mechanisms by which smoking affects OSAHS include changes in sleep architecture, neuromuscular dysfunction of the upper airway, frequent awakenings and enhancement in upper-airway inflammation [13]. Additionally, non-treated OSAHS has been correlated with increased smoking addiction [18,19]. Yet, there is a need for more large-scale studies, in order to clarify the relationship of these two disorders.

Apart from OSAHS, there are also sleep disorders that are not directly related to apneic episodes during sleep. Sleep behaviors, such as sleep talking, sleepwalking, sleep paralysis, night terrors, nightmares, restless sleep, bruxism, sleep-related eating disorder, restless legs and abnormal movements during sleep, are a nuisance for a significant part of the general population; however, there is little understanding about the pathogenesis of these disorders. Smoking has been proposed to be one of the etiologic factors for these disorders, particularly in the form of second-hand smoke exposure during pregnancy or early childhood [20,21]. In addition to that, smoking has been associated with possible REM behavior disorder (RBD) [22,23], sleep-related eating disorder [24] and sleep paralysis [25]. However, other studies failed to prove a relationship between smoking and parasomnias in general [26].

The aim of this study was to assess possible relations between smoking history and OSAHS-related symptoms, sleep study findings and co-morbidities. Additionally, this study aimed to evaluate other non-OSAHS-related sleep disorders, in patients visiting a sleep clinic, in order to investigate a holistic relationship between smoking and sleep disturbances.

## 2. Materials and Methods

The protocol of this cross-sectional study was approved by the local ethics committee (reference number: 965/290618) and all participants gave their written informed consent. All adult patients who visited the sleep clinic in our hospital from September 2010 to September 2020 and consented to participate were considered eligible and were included in the analysis. The patients were divided by their smoking history as current smokers (adults who have smoked 100 cigarettes in their lifetime and who currently smoke cigarettes), former smokers (adults who have smoked at least 100 cigarettes in their lifetime but who had quit smoking at the time of interview) and never smokers (adults who have never smoked or who have smoked less than 100 cigarettes in their lifetime), according to the definitions of the National Health Interview Survey (NHIS) of the US Centers for Disease Control and Prevention (CDC) [27]. Current smokers and former smokers were also grouped together, creating the group of ever smokers. Eventually, 4347 participants (1498 never smokers, 1480 former smokers and 1369 current smokers) were included in the analysis.

The baseline characteristics of the participants were recorded and included their age, gender, family status, body mass index (BMI), neck, waist and hip circumference, Malampati score, SaO_2_, heart rate and arterial blood pressure. The participants answered a questionnaire about their smoking status, i.e., their smoking history, the number of cigarettes smoked per day and the number of packyears. The medical history of the participants was also recorded (alcohol consumption, cardiovascular, respiratory, metabolic and psychiatric co-morbidities). In addition, questionnaires about night-time sleep and nap duration, sleep latency, questions regarding possible sleep disturbances (the existence of nightmares, sleep talking, abnormal movements, restless sleep or leg movements) and about OSA-related symptoms (dry mouth, morning fatigue, bad mood, headaches, heavy head, memory loss, dropping thing from hands, needing a passenger when driving to be kept awake, snoring frequency and loudness, choking–breathing pauses during sleep and night awakenings) were completed. Epworth sleepiness scale (ESS) [28], Berlin and STOP bang questionnaires [29,30], Athens insomnia scale (AIS) [31] and Rosenberg self-esteem scale [32] were also included

All patients that participated in the study were subjected to sleep studies: 186 underwent full polysomnography (PSG) (type 1 sleep study, including: electroencephalogram (EEG), electrooculogram (EOG), electromyogram (EMG), electrocardiogram (ECG), airflow, respiratory effort and oxygen saturation measurements) and the rest polygraphy (type 3 sleep study, including: respiratory movement, airflow, pulse rate and oxygen saturation measurements) [33]. The sleep studies were manually scored according to the American Academy of Sleep Medicine (AASM) criteria [34], by sleep technicians with more than 3 years of experience. Apnea hypopnea index (AHI) was used to evaluate OSA severity (no OSA: AHI < 5, mild OSA: AHI 5–15, moderate OSA: AHI 15–30, severe OSA: AHI > 30) [35,36].

Statistical analysis was performed using SPSS (version 20 IBM SPSS statistical software, Armonk, NY, USA). Continuous variables were presented as mean ± SD and categorical variables as number/total (%). *p* < 0.05 was accepted as statistically significant. To separate parametric from non-parametric variables, normality tests using the Kolmogorov–Smirnov test were performed. To detect significant differences between current, former and never smokers, a one-way ANOVA or a Kruskal–Wallis test was performed for parametric and non-parametric variables, respectively, followed by a post hoc analysis between pairs of groups, using the Bonferroni test or the Mann–Whitney U test for parametric and non-parametric variables, respectively. An independent samples *T* test or Mann–Whitney U test was performed for parametric and non-parametric variables, respectively, in order to detect significant differences between ever and never smokers. To detect significant differences for categorical variables, between current, former and never smokers or between ever and never smokers, a Chi-Square Test, using the Fisher’s exact test where appropriate, was performed. A post hoc analysis, using the same tests, was performed between the pairs of groups of current, former and never smokers. Finally, to compare AHI among possible answers regarding abnormal sleep behaviors between ever and never smokers, the same parametric or non-parametric tests for continuous variables were used, where appropriate, in the same way as previously described.

## 3. Results

Comparison between former and current smokers showed that former smokers had a history of consumption of a significantly greater number of cigarettes per day and pack/years and that former smokers had quit 4.36 ± 7.84 years before study enrollment. Although former smokers were significantly older than never smokers, no significant age differences were established between ever and never smokers (53.3 ± 12.7 years vs. 53.7 ± 14.9 years, *p* = 0.26). The group of ever smokers included significantly more males compared to never smokers (78.1% vs. 58.6%, *p* < 0.001) with significantly higher BMI and larger neck and waist, but not hip, circumference (Table 1). Ever smokers were consuming more alcohol (*p* < 0.001) and suffered more frequently from diabetes mellitus (16.9% vs. 14.0%, *p* = 0.012), coronary heart disease (13.2% vs. 6.2%, *p* < 0.001), acute myocardial infarction (3.6% vs. 1.1%, *p* < 0.001), hyperlipidemia (18.0% vs. 15.0%, *p* = 0.014) and pulmonary disease (10.0% vs. 5.3%, *p* < 0.001), while the opposite was true for hypothyroidism (10.0% vs. 16.5%, *p* < 0.001) (Table 2).

From the data from sleep questionnaires, never smokers presented significantly higher night-sleep duration compared to ever smokers (3.18 ± 0.78 h vs. 3.13 ± 0.77 h, *p* = 0.044), who, on the other hand, presented longer nap duration (2.95 ± 0.58 h vs. 2.86 ± 0.61 h, *p* < 0.001). Never smokers had higher self-esteem, with a significantly higher score in the Rosenberg self-esteem scale (22.2 ± 4.8 vs. 21.8 ± 4.8, *p* = 0.035). Berlin and STOP Bang questionnaires predicted higher risk for OSAHS in ever smokers compared to never smokers (87.1% vs. 84.6%, *p* = 0.024 and 96.2% vs. 93.0%, *p* < 0.001, respectively). However, there were no significant differences between the two groups regarding ESS and AIS (*p* = 0.13, *p* = 0.83, respectively). Yet, ever smokers, compared to never smokers, presented significantly more frequent episodes of sleep talking (30.8% vs. 26.6%, *p* = 0.004), abnormal movements (31.1% vs. 27.7%, *p* = 0.021), restless sleep (59.1% vs. 51.6%, *p* < 0.001) and leg movements (*p* = 0.002), especially the group of current smokers (Table 3) (*p* < 0.001 for all comparisons between never and current smokers in the post hoc analysis).

In terms of OSA-related symptoms, there were no differences between ever and never smokers in the presence of symptoms, such as dry mouth, morning fatigue, bad mood, memory loss or dropping things from hands. Never smokers declared significantly more frequent headaches and/or heavy head compared to ever smokers (*p* < 0.001 in both), while the opposite applied for the need of the presence of a passenger when driving to be kept awake (19.9% vs. 15.4%, *p* < 0.001). Ever smokers also presented significantly more frequent breathing pauses during sleep (*p* = 0.011), while their snoring was louder (*p* = 0.008), especially the current smokers (Table 4).

From the data collected from the sleep studies, ever smokers had significantly higher AHI and ODI compared to never smokers (34.4 ± 24.6 events/h vs. 31.7 ± 23.6 events/h, *p* < 0.001 and 33.9 ± 25.0 events/h vs. 31.0 ± 23.9 events/h, *p* < 0.001) and significantly lower mean SaO_2_ (91.8 ± 3.4% vs. 92.2 ± 3.9%, *p* < 0.001). Furthermore, they suffered more frequently from moderate and severe OSAHS compared to never smokers (50.3% vs. 46.9% and 26.2% vs. 24.8%, respectively), who were more frequently diagnosed with no or mild disease (13.1% vs. 12.7% and 15.3% vs. 10.8%, respectively, *p* < 0.001) (Table 5).

Finally, in both ever and never smokers who reported increased frequency of leg movements and other abnormal sleep behaviors, including sleep talking, abnormal movements and restless sleep, AHI was significantly higher (*p* < 0.001 in all of them), with the exception of nightmares (*p* = 0.52 in ever and *p* = 0.31 in never smokers) (Table 6).

## 4. Discussion

In this large-scale cross-sectional study, it was found that the severity of OSAHS was significantly greater in ever smokers compared to never smokers; Berlin and STOP Bang questionnaires predicted higher risk for OSAHS in ever smokers compared to never smokers. Additionally, there was a significant correlation between positive smoking history and sleep talking, and restless sleep and leg movements during sleep.

To the best of our knowledge, this is one of the largest observational studies—with a total of 4347 participants—which investigated the relationship between smoking and OSAHS. A recently published meta-analysis included a higher number of patients, but the data were derived from 13 distinct studies, and each one included a lower number of participants compared to ours [37]. Similar to previously published data, the present study also found that AHI was significantly higher in ever smokers compared to never smokers and that smoking is associated with OSA severity [9,13,37,38,39]. However, ESS and minimum SaO_2_ did not differ between these two groups, whereas ODI and mean SaO_2_ did [37]. Excessive daytime sleepiness was not found to differ between the two groups, which is in accordance with the fact that both groups presented similar prevalence in the majority of OSA-related symptoms, such as morning fatigue, bad mood, memory loss and falling asleep during reading.

Another interesting finding in the present research was that OSAHS patients in the ever-smoker group exhibited a significantly higher prevalence of co-morbidities, such as diabetes mellitus, hyperlipidemia, coronary disease, acute myocardial infarction and pulmonary disease. Previous studies have shown that untreated OSAHS, especially severe, significantly increases the incidence of diabetes mellitus, ischemic heart disease and acute myocardial infarction [40]. Therefore, smoking could contribute to the pathogenesis of these diseases, both directly, with its well-known mechanisms of action on the vascular endothelium, and indirectly, by increasing OSAHS severity.

Apart from OSAHS-related findings, this study also demonstrated that smoking had an impact on other non-OSAHS-related sleep parameters. Previous studies have shown that sleep quality might be worse in smokers, with increased sleep latency, higher prevalence of awakenings and difficulty in waking up [14,15,16,41]. In our study, sleep latency and reported awakenings did not differ significantly between ever and never smokers. However, night-sleep duration was significantly shorter in ever smokers, who presented longer nap duration during the daytime. These findings are in accordance with those of previous studies [42,43] in populations other than OSAHS, who had not been evaluated for sleep apnea with sleep studies. In addition to that, in the present study, abnormal sleep behaviors, such as sleep talking, abnormal movements, restless sleep and leg movements during sleep, were significantly more frequent in ever smokers and particularly in current smokers. This phenomenon has been observed, with second-hand smoke exposure during pregnancy or early childhood [20,21], or even with adult smoking [22,23,24,25], but not in all studies [26], none of which included OSAHS patients. Furthermore, abnormal sleep behaviors have also been related to increasing AHI [44,45,46]. Yet, to the best of our knowledge, our study is the first one to demonstrate that in a population of patients with OSAHS, there might be a significant correlation between positive smoking history and both abnormal sleep behaviors and increasing AHI. Thus, it is plausible to suggest that there might be a positive correlation between smoking history and abnormal sleep behaviors, not only directly but also indirectly, by increased AHI in ever smokers.

This study presents several limitations. It is a cross-sectional study and one cannot establish a cause-and-effect relationship between smoking and sleep disorders because the temporal sequence between the two cannot be determined. Furthermore, there were significant differences between ever and never smokers in baseline characteristics, such as BMI, neck and waist circumference, that might be responsible for the more severe presentation of OSAHS in these groups. Additional confounding factors contributing to more severe OSAHS in the group of ever smokers include increased alcohol consumption and a higher frequency of pulmonary and cardiovascular co-morbidities. On the other hand, cardiovascular disease may be the result of the more severe presentation of OSAHS, instead of the cause, creating a cause-and-effect loop. In any case, the population in our study represents a “real-life” patient group, visiting a sleep clinic, and the results should be interpreted under this spectrum. Another limitation is that full PSG was conducted in a minority of patients, while the majority was assessed with type 3 sleep studies, preventing a detailed evaluation of sleep architecture. Moreover, the presence of sleep behaviors was not based on objective means, but was made subjectively by the patients or their partners, leading to a potential recall bias.

Despite its limitations, the current study demonstrated a more severe presentation of OSAHS in smokers, with more frequent metabolic and cardiovascular co-morbidities, although it did not confirm the presence of the excessive daytime sleepiness of previous studies in this group of patients. More importantly, to the best of our knowledge, this is the first large-scale cross-sectional study that reported a significantly higher frequency of different sleep behaviors in smokers compared to non-smokers in patients with OSAHS. Hence, taking into consideration the findings of this study, the term “smoking obstructive sleep apnea” could be considered as a distinctive phenotype [38]. Future studies, more focused on additional sleep symptoms and disorders, are necessary in order to evaluate, in more detail, smoking-induced disturbed sleep patterns.

## Figures and Tables

**Table 1:** Baseline characteristics.

Characteristic		Smoking Status				Ever Smoker		
		Never Smoker	Former Smoker	Current Smoker	*p* (Value)	No	Yes	*p* (Value)
Age (years)		53.7 ± 14.9 (N = 1493)	57.5 ± 12.0 (N = 1478)	48.7 ± 11.7 (N = 1364)	<0.001	53.7 ± 14.9 (N = 1493)	53.3 ± 12.7 (N = 2842)	0.26
Gender	Female	619/1495 (41.4%)	322/1368 (23.5%)	301/1480 (20.3%)	<0.001	619/1495 (41.4%)	623/2848 (21.9%)	<0.001
	Male	876/1495 (58.6%)	1046/1368 (76.5%)	1179/1480 (79.7%)		876/1495 (58.6%)	2225/2848 (78.1%)	
Family status	Single	213/1213 (17.6%)	115/1199 (9.6%)	226/1157 (19.5%)	<0.001	213/1213 (17.6%)	341/2356 (14.5%)	0.013
	Married	969/1213 (79.9%)	1047/1199 (87.3%)	874/1157 (75.5%)		969/1213 (79.9%)	1921/2356 (81.5%)	
	Divorced	31/1213 (2.6%)	37/1199 (3.1%)	54/1157 (4.7%)		31/1213 (2.6%)	91/2356 (3.9%)	
Cigarettes per day		n/a	30.2 ± 19.9 (N = 1011)	21.5 ± 13.4 (N = 1345)	<0.001	n/a	25.3 ± 17.1 (N = 2356)	n/a
Packyears		n/a	42.6 ± 38.0 (N = 1011)	32.4 ± 25.0 (N = 1345)	<0.001	n/a	36.8 ± 31.6 (N = 2356)	n/a
BMI (kg/m^2^)		32.9 ± 7.3 (N = 1451)	33.8 ± 6.9 (N = 1445)	33.1 ± 7.2 (N = 1350)	0.002	32.9 ± 7.3 (N = 1451)	33.5 ± 7.1 (N = 2795)	0.024
Neck circumference (cm)		41.1 ± 7.1 (N = 959)	42.6 ± 6.3 (N = 956)	41.9 ± 6.8 (N = 921)	<0.001	41.1 ± 7.1 (N = 959)	42.2 ± 6.5 (N = 1877)	<0.001
Waist circumference (cm)		110.7 ± 18.0 (N = 820)	112.4 ± 18.3 (N = 828)	114.9 ± 16.2 (N = 793)	<0.001	110.7 ± 18.0 (N = 820)	113.7 ± 17.3 (N = 1621)	<0.001
Hip circumference (cm)		112.3 ± 15.4 (N = 776)	113.5 ± 13.8 (N = 784)	112.1 ± 15.9 (N = 751)	0.16	112.3 ± 15.4 (N = 776)	112.8 ± 14.9 (N = 1535)	0.49
Malampati score		2.7 ± 0.7 (N = 95)	2.8 ± 0.6 (N = 92)	2.8 ± 0.6 (N = 104)	0.64	2.7 ± 0.7 (N = 95)	2.8 ± 0.6 (N = 196)	0.39
SaO_2_ (%)		96.4 ± 2.2 (N = 981)	95.9 ± 2.9 (N = 999)	96.3 ± 1.8 (N = 937)	<0.001	96.4 ± 2.2 (N = 981)	96.1 ± 2.5 (N = 1936)	<0.001
Heart rate (beats/minute)		80.0 ± 13.5 (N = 980)	78.8 ± 13.0 (N = 996)	82.3 ± 13.0 (N = 933)	<0.001	80.0 ± 13.5 (N = 980)	80.5 ± 13.1 (N = 1929)	0.38
Systolic blood pressure (mmHg)		125.5 ± 17.1 (N = 273)	127.9 ± 15.5 (N = 312)	125.5 ± 16.8 (N = 262)	0.12	125.5 ± 17.1 (N = 273)	126.8 ± 16.2 (N = 574)	0.29
Diastolic blood pressure (mmHg)		76.3 ± 9.7 (N = 272)	77.5 ± 10.0 (N = 309)	77.2 ± 10.3 (N = 261)	0.34	76.3 ± 9.7 (N = 272)	77.4 ± 10.2 (N = 570)	0.16

N = number, n/a = not applicable, m = meters, Kg = kilograms, cm = centimeters, mmHg = millimeters of Mercury.

**Table 2:** Co-morbidities.

Co-Morbidity		Smoking Status				Ever Smoker		
		Never Smoker	Former Smoker	Current Smoker	*p* (Value)	No	Yes	*p* (Value)
Alcohol	Almost never	685/1469 (46.6%)	442/1452 (30.4%)	386/1343 (28.7%)	<0.001	685/1469 (46.6%)	828/2795 (29.6%)	<0.001
	A few times per month	685/1469 (46.6%)	829/1452 (57.1%)	729/1343 (54.3%)		685/1469 (46.6%)	1558/2795 (55.7%)	
	1–2 times per week	57/1469 (3.9%)	83/1452 (5.7%)	109/1343 (8.1%)		57/1469 (3.9%)	192/2795 (6.9%)	
	3–5 times per week	23/1469 (1.6%)	53/1452 (3.7%)	67/1343 (5.0%)		23/1469 (1.6%)	120/2795 (4.3%)	
	Every day	19/1469 (1.3%)	45/1452 (3.1%)	52/1343 (3.9%)		19/1469 (1.3%)	97/2795 (3.5%)	
Hypertension		630/1498 (42.1%)	683/1480 (46.1%)	450/1369 (32.9%)	<0.001	630/1498 (42.1%)	1133/2849 (39.8%)	0.14
Diabetes Mellitus		209/1498 (14.0%)	311/1480 (21.0%)	170/1369 (12.4%)	<0.001	209/1498 (14.0%)	481/2849 (16.9%)	0.012
Coronary disease		93/1498 (6.2%)	266/1480 (18.0%)	109/1369 (8.0%)	<0.001	93/1498 (6.2%)	375/2849 (13.2%)	<0.001
Acute myocardial infarction		17/1498 (1.1%)	68/1480 (4.6%)	33/1369 (2.4%)	<0.001	17/1498 (1.1%)	101/2849 (3.6%)	<0.001
Heart failure		12/1498 (0.8%)	19/1480 (1.3%)	5/1369 (0.4%)	0.026	12/1498 (0.8%)	24/2849 (0.8%)	0.89
Arrythmia		195/1498 (13.0%)	256/1480 (17.3%)	122/1369 (8.9%)	<0.001	195/1498 (13.0%)	378/2849 (13.3%)	0.82
Hyperlipidemia		225/1498 (15.0%)	309/1480 (20.9%)	203/1369 (14.8%)	<0.001	225/1498 (15.0%)	512/2849 (18.0%)	0.014
Ischemic stroke		41/1498 (2.7%)	47/1480 (3.2%)	39/1369 (2.9%)	0.76	41/1498 (2.7%)	86/2849 (3.0%)	0.60
Pulmonary disease		79/1498 (5.3%)	193/1480 (13.0%)	93/1369 (6.8%)	<0.001	79/1498 (5.3%)	286/2849 (10.0%)	<0.001
Hypothyroidism		247/1498 (16.5%)	168/1480 (11.4%)	117/1369 (8.6%)	<0.001	247/1498 (16.5%)	285/2849 (10.0%)	<0.001
Depression		27/1498 (1.8%)	31/1480 (2.1%)	45/1369 (3.3%)	0.023	27/1498 (1.8%)	76/2849 (2.7%)	0.08

**Table 3:** Sleep symptoms and sleep questionnaires according to the smoking status of the participants.

Characteristic		Smoking Status				Ever Smoker		
		Never Smoker	Former Smoker	Current Smoker	*p* (Value)	No	Yes	*p* (Value)
Epworth sleepiness scale		9.6 ± 4.7 (N = 1473)	9.9 ± 4.5 (N = 1459)	9.7 ± 4.7 (N = 1352)	0.23	9.6 ± 4.7 (N = 1473)	9.8 ± 4.6 (N = 2811)	0.13
Athens insomnia scale		17.1 ± 5.5 (N = 1241)	17.0 ± 5.6 (N = 1250)	17.2 ± 5.2 (N = 1181)	0.66	17.1 ± 5.5 (N = 1241)	17.1 ± 5.4 (N = 2431)	0.83
Rosenberg self-esteem scale		22.2 ± 4.8 (N = 758)	22.1 ± 4.8 (N = 788)	21.5 ± 4.8 (N = 806)	0.003	22.2 ± 4.8 (N = 758)	21.8 ± 4.8 (N = 1594)	0.035
Berlin questionnaire	Low risk	226/1469 (15.4%)	181/1452 (12.5%)	179/1344 (13.3%)	0.06	226/1469 (15.4%)	360/2796 (12.9%)	0.024
	High risk	1243/1469 (84.6%)	1271/1452 (87.5%)	1165/1344 (86.7%)		1243/1469 (84.6%)	2436/2796 (87.1%)	
Stop bang questionnaire	Low risk	66/939 (7.0%)	43/900 (4.8%)	27/938 (2.9%)	<0.001	66/939 (7.0%)	70/1838 (3.8%)	<0.001
	High risk	873/939 (93.0%)	857/900 (95.2%)	911/938 (97.1%)		873/939 (93.0%)	1768/1838 (96.2%)	
Nightmares		430/1498 (28.7%)	412/1480 (27.8%)	378/1369 (27.6%)	0.79	430/1498 (28.7%)	790/2849 (27.7%)	0.50
Sleep talking		398/1498 (26.6%)	421/1480 (28.5%)	456/1369 (33.3%)	<0.001	398/1498 (26.6%)	877/2849 (30.8%)	0.004
Abnormal movements during sleep		415/1498 (27.7%)	433/1480 (29.3%)	452/1369 (33.0%)	0.006	415/1498 (27.7%)	885/2849 (31.1%)	0.021
Restless sleep		773/1498 (51.6%)	872/1480 (58.9%)	811/1369 (59.2%)	<0.001	773/1498 (51.6%)	1683/2849 (59.1%)	<0.001
Legs movements	Do not know	60/1481 (4.1%)	58/1462 (4.0%)	54/1357 (4.0%)	<0.001	60/1481 (4.1%)	112/2819 (4.0%)	0.002
	Never	402/1481 (27.1%)	384/1462 (26.3%)	326/1357 (24.0%)		402/1481 (27.1%)	710/2819 (25.2%)	
	Rarely	93/1481 (6.3%)	91/1462 (6.2%)	105/1357 (7.7%)		93/1481 (6.3%)	196/2819 (7.0%)	
	Sometimes	447/1481 (30.2%)	405/1462 (27.7%)	327/1357 (24.1%)		447/1481 (30.2%)	732/2819 (26.0%)	
	Usually	407/1481 (27.5%)	440/1462 (30.1%)	439/1357 (32.4%)		407/1481 (27.5%)	879/2819 (31.2%)	
	Always	72/1481 (4.9%)	84/1462 (5.8%)	106/1357 (7.8%)		72/1481 (4.9%)	190/2819 (6.7%)	

**Table 4:** Sleep-apnea-related symptoms according to the smoking status of the participants.

Symptom		Smoking Status				Ever Smoker		
		Never Smoker	Former Smoker	Current Smoker	*p* (Value)	No	Yes	*p* (Value)
Dry mouth	Do not Know	2/1479 (0.1%)	4/1465 (0.3%)	2/1360 (0.2%)	0.74	2/1479 (0.1%)	6/2825 (0.2%)	0.55
	Almost never	411/1479 (27.8%)	423/1465 (28.9%)	388/1360 (28.5%)		411/1479 (27.8%)	811/2825 (28.7%)	
	1–2 times per month	14/1479 (1.0%)	12/1465 (0.8%)	6/1360 (0.4%)		14/1479 (1.0%)	18/2825 (0.6%)	
	1–2 times per week	19/1479 (1.3%)	28/1465 (1.9%)	24/1360 (1.8%)		19/1479 (1.3%)	52/2825 (1.8%)	
	3–4 times per week	38/1479 (2.6%)	38/1465 (2.6%)	29/1360 (2.1%)		38/1479 (2.6%)	67/2825 (2.4%)	
	Daily	995/1479 (67.3%)	960/1465 (65.5%)	911/1360 (67.0%)		995/1479 (67.3%)	1871/2825 (66.2%)	
Morning fatigue	Do not Know	1/1483 (0.1%)	4/1467 (0.3%)	2/1361 (0.2%)	0.035	1/1483 (0.1%)	6/2828 (0.2%)	0.38
	Almost never	416/1483 (28.1%)	468/1467 (31.9%)	369/1361 (27.1%)		416/1483 (28.1%)	837/2828 (29.6%)	
	1–2 times per month	15/1483 (1.0%)	21/1467 (1.4%)	16/1361 (1.2%)		15/1483 (1.0%)	37/2828 (1.3%)	
	1–2 times per week	47/1483 (3.2%)	39/1467 (2.7%)	29/1361 (2.1%)		47/1483 (3.2%)	68/2828 (2.4%)	
	3–4 times per week	63/1483 (4.3%)	64/1467 (4.4%)	46/1361 (3.4%)		63/1483 (4.3%)	110/2828 (3.9%)	
	Daily	941/1483 (63.5%)	871/1467 (59.4%)	899/1361 (66.1%)		941/1483 (63.5%)	1770/2828 (62.6%)	
Bad mood	Almost never	414/1483 (27.9%)	469/1467 (32.0%)	369/1361 (27.1%)	0.034	414/1483 (27.9%)	838/2828 (29.6%)	0.60
	1–2 times per month	17/1483 (1.2%)	21/1467 (1.4%)	15/1361 (1.1%)		17/1483 (1.2%)	36/2828 (1.3%)	
	1–2 times per week	48/1483 (3.2%)	38/1467 (2.6%)	35/1361 (2.6%)		48/1483 (3.2%)	73/2828 (2.6%)	
	3–4 times per week	70/1483 (4.7%)	67/1467 (4.6%)	53/1361 (3.9%)		70/1483 (4.7%)	120/2828 (4.2%)	
	Almost daily	933/1483 (62.9%)	868/1467 (59.2%)	889/1361 (65.3%)		933/1483 (62.9%)	1757/2828 (62.1%)	
	Daily	1/1483 (0.1%)	4/1467 (0.3%)	0/1361 (0.0%)		1/1483 (0.1%)	4/2828 (0.1%)	
Headache	Do not Know	3/1483 (0.2%)	1/1467 (0.1%)	0/1360 (0.0%)	<0.001	3/1483 (0.2%)	1/2827 (0.0%)	<0.001
	Almost never	841/1483 (56.7%)	942/1467 (64.2%)	845/1360 (62.1%)		841/1483 (56.7%)	1787/2827 (63.2%)	
	1–2 times per month	68/1483 (4.6%)	52/1467 (3.6%)	41/1360 (3.0%)		68/1483 (4.6%)	93/2827 (3.3%)	
	1–2 times per week	75/1483 (5.1%)	73/1467 (5.0%)	90/1360 (6.6%)		75/1483 (5.1%)	163/2827 (5.8%)	
	3–4 times per week	109/1483 (7.4%)	81/1467 (5.5%)	69/1360 (5.1%)		109/1483 (7.4%)	150/2827 (5.3%)	
	Daily	387/1483 (26.1%)	318/1467 (21.7%)	315/1360 (23.2%)		387/1483 (26.1%)	633/2827 (22.4%)	
Heavy head	Do not Know	2/1482 (0.1%)	2/1467 (0.1%)	2/1361 (0.2%)	0.001	2/1482 (0.1%)	4/2828 (0.1%)	<0.001
	Almost never	827/1482 (55.8%)	928/1467 (63.3%)	841/1361 (61.8%)		827/1482 (55.8%)	1769/2828 (62.6%)	
	1–2 times per month	67/1482 (4.5%)	47/1467 (3.2%)	34/1361 (2.5%)		67/1482 (4.5%)	81/2828 (2.9%)	
	1–2 times per week	73/1482 (4.9%)	67/1467 (4.6%)	77/1361 (5.7%)		73/1482 (4.9%)	144/2828 (5.1%)	
	3–4 times per week	111/1482 (7.5%)	89/1467 (6.1%)	68/1361 (5.0%)		111/1482 (7.5%)	157/2828 (5.6%)	
	Daily	402/1482 (27.1%)	334/1467 (22.8%)	339/1361 (24.9%)		402/1482 (27.1%)	673/2828 (23.8%)	
Need a passenger when driving to be kept awake		231/1498 (15.4%)	299/1480 (20.2%)	269/1369 (19.7%)	0.001	231/1498 (15.4%)	568/2849 (19.9%)	<0.001
Snoring frequency	Never	15/1498 (1.0%)	14/1480 (1.0%)	9/1369 (0.7%)	0.31	15/1498 (1.0%)	23/2849 (0.8%)	0.20
	Almost never	46/1498 (3.1%)	44/1480 (3.0%)	25/1369 (1.8%)		46/1498 (3.1%)	69/2849 (2.4%)	
	1–2 times per month	5/1498 (0.3%)	3/1480 (0.2%)	3/1369 (0.2%)		5/1498 (0.3%)	6/2849 (0.2%)	
	1–2 times per week	9/1498 (0.6%)	3/1480 (0.2%)	6/1369 (0.4%)		9/1498 (0.6%)	9/2849 (0.3%)	
	3–4 times per week	13/1498 (0.9%)	7/1480 (0.5%)	7/1369 (0.5%)		13/1498 (0.9%)	14/2849 (0.5%)	
	Every night	1393/1498 (93.0%)	1385/1480 (93.6%)	1296/1369 (94.7%)		1393/1498 (93.0%)	2681/2849 (94.1%)	
	Many times per night	17/1498 (1.1%)	24/1480 (1.6%)	23/1369 (1.7%)		17/1498 (1.1%)	47/2849 (1.7%)	
Breathing pauses during sleep	Almost never	171/1479 (11.6%)	137/1463 (9.4%)	123/1357 (9.1%)	0.06	171/1479 (11.6%)	260/2820 (9.2%)	0.011
	1–2 times per month	7/1479 (0.5%)	6/1463 (0.4%)	4/1357 (0.3%)		7/1479 (0.5%)	10/2820 (0.4%)	
	1–2 times per week	15/1479 (1.0%)	9/1463 (0.6%)	14/1357 (1.0%)		15/1479 (1.0%)	23/2820 (0.8%)	
	3–4 times per week	28/1479 (1.9%)	16/1463 (1.1%)	21/1357 (1.6%)		28/1479 (1.9%)	37/2820 (1.3%)	
	Every night	1056/1479 (71.4%)	1125/1463 (76.9%)	1036/1357 (76.4%)		1056/1479 (71.4%)	2161/2820 (76.6%)	
	Many times per night	202/1479 (13.7%)	170/1463 (11.6%)	159/1357 (11.7%)		202/1479 (13.7%)	329/2820 (11.7%)	
Night awakenings	Almost never	315/1477 (21.3%)	255/1466 (17.4%)	365/1357 (26.9%)	<0.001	315/1477 (21.3%)	620/2823 (22.0%)	0.51
	1–2 times per month	12/1477 (0.8%)	16/1466 (1.1%)	16/1357 (1.2%)		12/1477 (0.8%)	32/2823 (1.1%)	
	1–2 times per week	49/1477 (3.3%)	35/1466 (2.4%)	41/1357 (3.0%)		49/1477 (3.3%)	76/2823 (2.7%)	
	3–4 times per week	1018/1477 (68.9%)	1074/1466 (73.3%)	881/1357 (64.9%)		1018/1477 (68.9%)	1955/2823 (69.3%)	
	Every night	83/1477 (5.6%)	86/1466 (5.9%)	54/1357 (4.0%)		83/1477 (5.6%)	140/2823 (5.0%)	

**Table 5:** Sleep study parameters according to the smoking status of the participants.

Parameter		Smoking Status				Ever Smoker		
		Never Smoker	Former Smoker	Current Smoker	*p* (Value)	No	Yes	*p* (Value)
Total sleep time (minutes)		270.1 ± 73.0 (N = 70)	239.3 ± 81.6 (N = 63)	248.3 ± 82.2 (N = 70)	0.07	270.1 ± 73.0 (N = 70)	244.0 ± 81.8 (N = 133)	0.022
% REM sleep time (%)		25.1 ± 14.0 (N = 63)	24.2 ± 14.5 (N = 58)	20.8 ± 12.1 (N = 65)	0.17	25.1 ± 14.0 (N = 63)	22.4 ± 13.4 (N = 123)	0.20
% Non-REM sleep time (%)		74.9 ± 14.0 (N = 63)	75.8 ± 14.5 (N = 58)	79.2 ± 12.1 (N = 65)	0.17	74.9 ± 14.0 (N = 63)	77.6 ± 13.4 (N = 123)	0.20
Night sleep duration (hours)		3.18 ± 0.78 (N = 1482)	3.15 ± 0.79 (N = 1465)	3.12 ± 0.76 (N = 1361)	0.06	3.18 ± 0.78 (N = 1482)	3.13 ± 0.77 (N = 2826)	0.044
Sleep latency (minutes)		12.19 ± 1.20 (N = 1480)	12.11 ± 1.16 (N = 1463)	12.12 ± 1.17 (N = 1359)	0.17	12.19 ± 1.20 (N = 1480)	12.12 ± 1.16 (N = 2822)	0.07
Nap duration (hours)		2.86 ± 0.61 (N = 895)	2.92 ± 0.59 (N = 975)	2.99 ± 0.56 (N = 806)	<0.001	2.86 ± 0.61 (N = 895)	2.95 ± 0.58 (N = 1781)	<0.001
AHI (events/hour)		31.7 ± 23.6 (N = 1498)	35.5 ± 23.8 (N = 1480)	33.3 ± 25.4 (N = 1369)	<0.001	31.7 ± 23.6 (N = 1498)	34.4 ± 24.6 (N = 2849)	<0.001
Central apneas (events/hour)		0.7 ± 3.3 (N = 1488)	0.7 ± 2.6 (N = 1468)	0.5 ± 2.3 (N = 1355)	0.05	0.7 ± 3.3 (N = 1488)	0.6 ± 2.5 (N = 2823)	0.26
Mean SaO_2_ (%)		92.2 ± 3.9 (N = 1498)	91.6 ± 3.4 (N = 1480)	91.9 ± 3.3 (N = 1369)	<0.001	92.2 ± 3.9 (N = 1498)	91.8 ± 3.4 (N = 2849)	<0.001
Minimum SaO_2_ (%)		79.1 ± 9.8 (N = 1496)	78.0 ± 9.6 (N = 1477)	79.1 ± 9.5 (N = 1369)	0.001	79.1 ± 9.8 (N = 1496)	78.5 ± 9.6 (N = 2846)	0.08
ODI (events/hour)		31.0 ± 23.9 (N = 1498)	35.1 ± 24.6 (N = 1477)	32.7 ± 25.3 (N = 1368)	<0.001	31.0 ± 23.9 (N = 1498)	33.9 ± 25.0 (N = 2845)	<0.001
Mean apnea duration (seconds)		22.1 ± 7.8 (N = 1201)	22.3 ± 7.1 (N = 1197)	21.6 ± 7.5 (N = 1127)	0.06	22.1 ± 7.8 (N = 1201)	22.0 ± 7.3 (N = 2324)	0.55
Maximum apnea duration (seconds)		47.5 ± 24.0 (N = 985)	50.1 ± 24.6 (N = 991)	47.0 ± 23.0 (N = 936)	0.009	47.5 ± 24.0 (N = 985)	48.6 ± 23.9 (N = 1927)	0.22
OSA diagnosis	Absent (AHI < 5)	229/1498 (15.3%)	124/1480 (8.4%)	184/1369 (13.4%)	<0.001	229/1498 (15.3%)	308/2849 (10.8%)	<0.001
	Mild (AHI 5–15)	196/1498 (13.1%)	176/1480 (11.9%)	186/1369 (13.6%)		196/1498 (13.1%)	362/2849 (12.7%)	
	Moderate (AHI 15–30)	371/1498 (24.8%)	394/1480 (26.6%)	352/1369 (25.7%)		371/1498 (24.8%)	746/2849 (26.2%)	
	Severe (AHI > 30)	702/1498 (46.9%)	786/1480 (53.1%)	647/1369 (47.3%)		702/1498 (46.9%)	1433/2849 (50.3%)	

N = number, REM = rapid eye movement, AHI = apnea hypopnea index, ODI = oxygen desaturation index, OSA = obstructive sleep apnea–hypopnea syndrome.

**Table 6:** Comparison of AHI and abnormal sleep behaviors according to smoking history.

Abnormal Sleep Behavior		Never Smokers		Ever Smoker	
		AHI	*p* (Value)	AHI	*p* (Value)
Nightmares	Yes	32.7 ± 23.6 (N = 430)	0.31	34.9 ± 25.3 (N = 790)	0.52
	No	31.3 ± 23.6 (N = 1068)		34.2 ± 24.3 (N = 2059)	
Sleep talking	Yes	36.9 ± 24.8 (N = 398)	<0.001	38.6 ± 25.9 (N = 877)	<0.001
	No	29.8 ± 22.9 (N = 1100)		32.6 ± 23.7 (N = 1972)	
Abnormal movements during sleep	Yes	35.4 ± 24.6 (N = 415)	<0.001	38.3 ± 25.9 (N = 885)	<0.001
	No	30.3 ± 23.1 (N = 1083)		32.7 ± 23.8 (N = 1964)	
Restless sleep	Yes	34.4 ± 24.3 (N = 773)	<0.001	36.6 ± 25.1 (N = 1683)	<0.001
	No	28.9 ± 22.6 (N = 725)		31.3 ± 23.6 (N = 1166)	
Legs movements	Do not know	24.5 ± 20.4 (N = 60)	<0.001	28.8 ± 24.5 (N = 112)	<0.001
	Never	27.1 ± 23.6 (N = 402)		31.0 ± 24.2 (N = 710)	
	Rarely	29.5 ± 22.7 (N = 93)		33.8 ± 23.9 (N = 196)	
	Sometimes	33.6 ± 23.5 (N = 447)		34.3 ± 22.7 (N = 732)	
	Usually	34.1 ± 22.7 (N = 407)		36.6 ± 25.3 (N = 879)	
	Always	41.6 ± 27.7 (N = 72)		41.8 ± 27.6 (N = 190)	

## Data Availability

Data used in the present study are available upon request from the corresponding author.
